# Report of the Committee on Surgery

**Published:** 1849-02

**Authors:** 


### MEDICAL EXAMINER,

AND

### RECORD OF MEDICAL SCIENCE.

NEW SERIES.—NO. L.—FEBRUARY, 1849.

### ORIGINAL COMMUNICATIONS.

[The Editors of the Medical Examiner publish below the entire Report of the Committee on Surgery, made to the American Medical Association at their last meeting, with the consent of its author, Dr. Norris; deeming its value sufficient apology for the exclusion of several communications, which, however, will appear in their next number.

Their motive in doing this is, that the Report may have a more general circulation among their readers than the comparatively limited distribution of the "Transactions" would give it.]

### REPORT OF THE COMMITTEE ON SURGERY.

In presenting the Report on Surgery, the committee beg leave to state, that in entering upon their duties, they felt themselves under some embarrassment from the terms in which those duties are defined. By our regulations they are directed to prepare a report on all the important improvements in the management of surgical diseases effected in America during the year. Had they confined themselves to the letter of this requisition, their task would have been a light one. Neither brilliant discoveries, nor any extraordinary improvements in the practice of surgery, have marked the past year. Many suggestive changes indeed are to be found—they are never failing—but all who devote themselves to the treatment of disease are aware how few of these bear the test either of experience or examination.

Though remarkable, as we have said, by no great discovery or very important improvement, yet the past year gives evidence of substantial progress, and in the estimation of your committee, this progress is evinced in nothing so much as in the desire which is everywhere shown to determine the actual value of operative procedures. On this account they have deemed it best to incorporate in their report, along with a notice of the improvements which are known to them to have either originated or been adopted in this country within the year, such results of surgical operations when done on a large scale, as they have been enabled to collect. In pursuing this course, the report necessarily becomes somewhat retrospective, and though the committee, by so doing, have not observed the letter of the law, they, nevertheless, have endeavoured to catch its spirit by seeking after materials from our own practitioners, and dwelling chiefly on improvements not yet generally adopted among us.

In order to collect matter for this report, the committee issued a circular letter, soliciting information in regard to some points of general interest to the profession, as well as of improvements in practice, expecting by this means to obtain facts and statistical data, which, when arranged and classified, might lead to interesting conclusions. So little, however, has been communicated to them, as not to be available for the purposes originally contemplated. Your committee cannot here withhold the expression of a hope that the future Surgical Reports to this Association may be made a repository for the statistical results of operations and modes of treatment, and that they may be more freely communicated than has yet been done.

It is only by collecting together a large number of facts that general conclusions at all approaching to accuracy can be attained, in addition to which, when drawn from the several sections of our widely extended country, as they might readily be, through the medium of a society such as this, they would allow of a comparison of the methods of treatment pursued by different practitioners and institutions, and might shed much light upon the effects of climate, as well as point out the greater or less frequency of particular surgical diseases in various localities.

To arrive, however, at accurate results from statistics, not only the records of several consecutive years are required, but they must also include all the cases of the disease, or operation treated of, which occur in the practice of the institution or surgeon, from which they emanate. They would specify the subjects of fractures and luxations, of amputations, of operations for stone, aneurism, cancer, hernia and cataract, as particularly worthy of statistical investigation.

Among the subjects of inquiry which presented themselves to the committee, none appeared to them more worthy of present attention than those of lithotomy and lithotrity; for, in addition to their practical bearing, it seems to be peculiarly fit at this time, when the attention of our brethren in Europe is again awakened to them, as it has been by the recent discussion of their merits and faults, in a learned body in Paris, that some account of the results of operations done for the relief of stone in the United States should be made known.

The causes which give rise to stone, and the relative frequency or rarity with which it is found in the different parts of our continent, are matters of much interest, and endeavours were made to gain intelligence for this report in regard to them, hoping thereby that something might be elicited to explain the probable local causes of its production. In this, however, they have been disappointed, and until an extensive series of observations can be gathered from the different sections of the country, no accurate knowledge on the subject will be obtained. Deprived as we thus are of any precise information on the matter, it may not be amiss to state, as the general impression of medical men among us, that calculus is rare in New England, more common in the Middle and Southern States, and that it is much more frequently met with in the Valley of the Mississippi than in any other portion of the country. Negroes are thought to be rarely the subjects of it, while in Canada it is said to be not uncommon. For the cure of stone, the cutting operation still continues to be that mostly resorted to, and the lateral operation with the gorget is believed to be the procedure most commonly employed. Nor is this to be wondered at, when the prepossession in its favour, derived from our earliest teachers, joined to the ease and rapidity with which the operation is done, and the fair results which usually follow it, are considered, added to which the unprecedented success that continues to be furnished by it in the hands of the eminent Professor of Surgery, in the Transylvania University, tends perhaps not a little still to popularize it among American surgeons.

In the last account* of the practice of Dr. Dudley which has

* Western Lancet, 1846.

reached us, it is stated that up to the beginning of 1846 he had operated upon 185 cases of stone, of which number 180 are reported as successful. This remarkable result, according to Dr. Bush, cannot be attributed to any selection of cases on the part of the operator, since out of 188 subjects presented to him, 185 were cut. Dr. B., who furnishes this report, ascribes these results to the thorough preparation of the general system made by Dr. Dudley, preparatory to the operation, an account of which was detailed some years since, in a paper published by him in the Transylvania Journal, and which we can only here refer to.

From communications that have been made to the committee, it appears that Dr. Marsh, of Albany, has operated by the lateral method seven times, all of which were successful.

Dr. Mettauer, of Virginia, states that he has operated by lithotomy on seventy-three cases of calculus, two of which proved fatal. One from prostatic hemorrhage, and the other from the occurrence of spasm of the ileum.

Dr. Jno. C. Warren has operated upon thirty cases, of whom two died; one of these lost his life by an error in diet, the other had a purulent effusion, owing to the great size of the stone, and the force required to extract it. The mode of operating in his fifteen first cases, was by the lateral incision and the gorget. In the thirteen following, by the knife, and in the three last by the bi-lateral method.

Dr. Eve, of Georgia, has operated eight times, including one female, all of which were successful. Dr. Mussey, of Cincinnati, informs us that he has cut thirty-two patients for stone, all of which cases have been successful but two.

From the Pennsylvania Hospital your committee have procured a tabular statement, which is herewith submitted as a part of their report, of all the patients cut in that Institution from its foundation in 1752 to the 1st of May, 1848, which, though in some respects imperfect, is nevertheless valuable, as exhibiting the largest mass of experience in calculus, which has yet been furnished by any American Institution.

| NO. | NAME. | ADMISSION. | DISCHARGED. | RESULT. |
|---|---|---|---|---|
| 1 | Ann Fust | October 22, 1756 | January 12, 1757 | Cured |
| 2 | James Miller | August 29, 1759 | October 10, 1759 | Cured |
| 3 | James Clark | April 14, 1763 | September 5, 1765 | Cured |
| 4 | James Child | May 4, 1765 | June 19, 1765 | Cured |
| 5 | John Harper | August 23, 1765 | October 16, 1765 | Cured |
| 6 | Jeremiah Tracy | October 2, 1765 | April 2, 1766 | Cured |
| 7 | Ann Coyle | June 27, 1782 | July 10, 1782 | Cured |
| 8 | Michael Fisher | November 10, 1786 | Dec. 12, 1786 | Cured |
| 9 | Jacob Felkner | November 29, 1788 | April 30, 1789 | Cured |
| 10 | James Bennet | September 1, 1792 | November 28, 1792 | Cured |
| 11 | James Fox | March 20, 1798 | April 7, 1798 | Cured |
| 12 | Abner Lamb | January 1, 1800 | January 21, 1800 | Died |
| 13 | James Shaw | August 31, 1801 | Sept. 31, 1802 | Died |
| 14 | John Garraghan | May 13, 1802 | June 16, 1802 | Cured |
| 15 | Isaac Vanderwalker | June 6, 1804 | August 22, 1804 | Cured |
| 16 | John Brebaker | May 9, 1809 | June 29, 1809 | Died |
| 17 | Joseph Bently | November 6, 1809 | June 15, 1810 | Cured |
| 18 | Thomas M'Dowell | March 27, 1810 | April 23, 1810 | Cured |
| 19 | George Wall | November 7, 1810 | February 7, 1811 | Cured |
| 20 | John Brown | November 13, 1811 | January 25, 1812 | Cured |
| 21 | Francis Welsh | February 22, 1812 | April 25, 1812 | Cured |
| 22 | Wm. P. Price | April 29, 1813 | May 15, 1813 | Died |
| 23 | Needham Bryan | May 12, 1813 | August 4, 1813 | Cured |
| 24 | Nathan Cattell | November 4, 1813 | January 22, 1814 | Cured |
| 25 | Gideon Goodwin | September 21, 1814 | Dec. 10, 1814 | Cured |
| 26 | Shadrach Mears | June 17, 1815 | August 21, 1815 | Cured |
| 27 | James Parker | July 6, 1816 | August 1, 1816 | Died |
| 28 | Jane Maise | September 1, 1816 | Nov. 15, 1816 | Cured |
| 29 | Claiborne Laughlin | June 21, 1817 | August 1, 1817 | Cured |
| 30 | Thomas Allen | June 22, 1817 | August 30, 1817 | Cured |
| 31 | Isabella Berry | June 29, 1818 | August 5, 1818 | Cured |
| 32 | James Moss | October 14, 1818 | January 26, 1819 | Cured |
| 33 | Richard Harris | December 5, 1818 | April 15, 1819 | Rel'vd |
| 34 | Penrose Fuhr. | December 7, 1819 | February 5, 1820 | Cured |
| 35 | Samuel J. Herron | December 8, 1819 | January 31, 1820 | Cured |
| 36 | Isaiah Baptiste | September 9, 1823 | October 25, 1823 | Cured |
| 37 | Edward Robuck | September 23, 1823 | January 7, 1824 | Cured |
| 38 | Samuel Austin | February 22, 1824 | April 5, 1824 | Died |
| 39 | Osmin Harris | December 30, 1824 | February, 1825 | Cured |
| 40 | Abraham Margerum | November 14, 1825 | January 2, 1826 | Cured |
| 41 | James Barber | April 14, 1826 | June 9, 1826 | Cured |
| 42 | John Chandler | July 21, 1828 | Nov. 15, 1828 | Cured |
| 43 | Samuel Suter | December 20, 1828 | Dec. 29, 1828 | Died |
| 44 | Charles Lex | January 21, 1829 | March 28, 1829 | Cured |
| 45 | Samuel M'Donald | April 2, 1829 | July 6, 1829 | Cured |
| 46 | Michael Engles | October 24, 1829 | Nov. 28, 1829 | Died |
| 47 | Robert Fry | September 23, 1829 | April 3, 1829 | Cured |

| NO. | NAME. | AGE. | ADMISSION. | DISCHARGED. | RESULT. |
|---|---|---|---|---|---|
| 48 | William Eastwood | 6 | April 28, 1832 | June 2, 1832 | Cured |
| 48 | Houston Sigman | 4 | Sept. 10, 1832 | Nov. 1, 1832 | Cured |
| 50 | Joseph Purlz | 3 | October 1, 1832 | October 28, 1832 | Died |
| 51 | Grayson Nelson | 14 | April 30, 1833 | July 3, 1833 | Cured |
| 52 | Henry Thorp | 19 | Nov. 7, 1833 | January 1, 1834 | Cured |
| 53 | Peter Spyers | 17 | Feb. 8, 1834 | July 19, 1834 | Cured |
| 54 | Daniel Gillan | 6 | May 31, 1835 | August 5, 1835 | Cured |
| 55 | James Driver | 16 | June 14, 1835 | July 29, 1835 | Cured |
| 56 | Stephen Black | 6 | Dec. 22, 1835 | Feb. 9, 1836 | Cured |
| 57 | Ellen Clincy | 10 | Feb. 29, 1836 | May 11, 1836 | Cured |
| 58 | Robert Thomson | 24 | March 17, 1836 | June 16, 1836 | Cured |
| 59 | William M'Elroy | 12 | January 14, 1836 | March 4, 1837 | Cured |
| 60 | Samuel Darby | 16 | April 3, 1836 | July 18, 1836 | Cured |
| 61 | Anthony Stresler | 43 | October 25, 1837 | January 23, 1838 | Cured |
| 62 | Bernard M'Kenna | 7 | Dec. 15, 1838 | Feb. 22, 1839 | Cured |
| 63 | John Hughes | 4 | January 19, 1839 | Feb. 6, 1839 | Cured |
| 64 | John Ransley | 2 | March 20, 1839 | June 6, 1839 | Cured |
| 65 | John Hughes, 2d | 5 | Nov. 11, 1839 | Dec. 4, 1839 | Cured |
| 66 | John Ramsey | 3 | January 8, 1840 | Feb. 22, 1840 | Cured |
| 67 | W. R. Patterson | 3 | May 5, 1840 | June 5, 1840 | Cured |
| 68 | William Bradley | 8 | Nov. 11, 1840 | May 20, 1840 | Cured |
| 69 | Thomas Carlin | 4 | Dec. 30, 1840 | Feb. 6, 1841 | Cured |
| 70 | James Wharton | 4 | January 10, 1842 | April 14, 1842 | Cured |
| 71 | Morgan Morgan | 5 | August 27, 1842 | October 24, 1842 | Cured |
| 72 | John M'Intyre | 3 | Sept. 21, 1842 | March 11, 1842 | Cured |
| 73 | John M'Cônnell | 3 | May, 1843 | July 1, 1843 | Cured |
| 74 | William Houston | 3 | May, 1843 | June 21, 1843 | Cured |
| 75 | Henry Huey | 5 | Sept. 19, 1843 | October 11, 1843 | Cured |
| 76 | C. C. Goldsborough | 21 | October 31, 1843 | January 15, 1844 | Cured |
| 77 | Thomas Hibbert | 3 | October 22, 1844 | Dec. 2, 1844 | Cured |
| 78 | Samuel Jarvis | 4 | Dec. 2, 1844 | Dec. 13, 1844 | Died |
| 79 | John O'Neill | 11 | Dec. 17, 1844 | Feb. 5, 1845 | Cured |
| 80 | Charles Ross | 41 | August 18, 1845 | October 22, 1845 | Cured |
| 81 | James Furphy | 4 | Sept. 17, 1845 | Nov. 8, 1845 | Cured |
| 82 | John Sharkey | 14 | Sept. 18, 1847 | October 29, 1847 | Cured |
| 83 | John Beck | 10 | January 26, 1848 | May 3, 1848 | Cured |

From this table, it appears that during the period mentioned, 83 cases underwent the operation of lithotomy, which, it is believed, was invariably by the lateral method, and except in a few instances of very young children, by means of the gorget. Of this number, 72 were cured, 10 died, and 1 is set down as relieved.

A few among us have resorted to the bi-lateral method, and within a few years the profession have been favoured with valuable papers on modifications of it by Drs. Warren and Stevens. So far as your committee can ascertain, the first operation in our country by this method was performed by Dr. Wm. Ashmead, of Philadelphia, in 1832, nearly eight years after it was brought prominently into notice by Dupuytren at the Hotel Dieu of Paris. The case proved successful, and in that and the succeeding years, the same gentlemen operated upon three other patients. Dr. Ogier, of Charleston, repeated the operation in 1835, without any knowledge of its having been previously done in the country, and since that period it is known to your committee to have been practiced by Dr. Stevens, Eve, the Warrens, Mussey, May, Watson, Hoffman, Post and Pancoast.

Lithotripsy, too, continues to have its advocates, and though during the past year no extended notice of it has been met with, yet it is not to be inferred that it is without warm advocates, or fails to occupy the attention of our practitioners and teachers. We know, on the contrary, that earnest endeavours are still making to relieve calculous patients by this means, and have reason to think that in portions of the country the operators by this method may be said to be on the increase.

It was intended to have included in this Report, a brief history of the introduction, progress and present condition of the crushing operation among us, accompanied with an extensive statistical table of cases of lithotripsy, which would have permitted of some comparison being made between its results, and those derived from the cutting operation. In consequence, however, of the lamented death of one of their colleagues, Dr. Randolph, who had engaged to furnish important materials and aid in this inquiry, the committee have been unable to accomplish their design. That gentleman, as is well known, was one of the earliest who adopted the method in our country, and by his exertions in teaching, and skill in the performance of the operation, did much to introduce it generally among us, and it is to be regretted that the fruits of his extensive experience, and a statement of his reverses and success, which latter is said upon competent authority never to have been excelled, had not been completed by him.

As indicative of an actual advance in the science of surgery, there is no subject which it more gratifies your committee to notice, than that of the treatment of aneurism by compression. Compression of the vessel between the aneurismal tumour and the heart, it is well known, was long ago employed, and examples of true aneurism of the lower extremity, radically cured by this means, are recorded; yet as the principle on which the treatment should be conducted was not then understood, much distress was occasioned by it, many failures occurred, and the practice fell into disuse. Scarpa emitted the opinion that it was by exciting adhesive inflammation in the internal coat of the vessel, that pressure effected the cure, and the same doctrine was afterwards held by our own experimenter, Jameson; but the recent and accurate observations of the Dublin surgeons, particularly of Mr. Bellingham, show that obliteration of the vessel by its inflammation and consequent effusion of lymph is not requisite for the cure, and have, as we think, satisfactorily proved, that for the cure of aneurism by compression above the sac, an absolute interruption to the circulation through the vessel is not demanded—the process of cure, when it occurs, being identical with that by which nature sometimes spontaneously effects it; viz., the gradual deposition of the fibrine of the blood in the sac until it is completely filled up, and no longer permits the entrance of that fluid. The practical deduction from this principle is, that the pressure need not be so great as entirely to interrupt the circulation at the point compressed, and that in fact our object may be attained by simply diminishing the current, and thus favouring the deposit.

In 27 cases, which are related in the valuable communication of Mr. Bellingham, from the practice of seventeen surgeons, 24 were cured. One died suddenly from disease of the heart, forty-eight hours after pressure had been removed, all pulsation in the aneurism having ceased. In another case the operation was done at the request of the patient after the pressure had been continued for a fortnight, and in the third, pulsation continuing some time after compression was resorted to, a galvanic current was passed through the sac, and was followed by erysipelas and death.

The ages of the patients operated on, varied from twenty-three to fifty-five years. The greatest length of time required for the cure in the above 24 cases was 106 days; the shortest, two days. The average duration of treatment was nearly thirty-nine days. Of the whole number of cases, three were femoral, and the rest popliteal aneurisms, and the case followed by death, as well as those in which the treatment failed, belonged to this latter class.

As to the instruments used in applying pressure, their shape and construction are matters of comparatively little importance. The essential points in an instrument for compression are: that it should admit of being readily applied: that its principle should be so simple as to be understood by the patient, and that it should effect the object intended with as little inconvenience as possible. A broad soft pad, Mr. Bellingham thinks, will generally be found to answer best, and the counter-pressure ought to be distributed over a large surface. As to the sites at which the pressure may be applied on the lower limb, either the point where the artery crosses the horizontal ramus of the pubis; between Poupart's ligament and the point at which the saphena joins the femoral vein; from the middle third of the thigh, down to the opening through which the artery passes to the back of the limb, are any of them eligible situations for it. In regard to the degree of compression, it at first ought always to be light; after a time, when tolerance is established, it may be increased to the degree we consider necessary; but, as before observed, Mr. Bellingham thinks it need never be so great as to interrupt completely the circulation in the artery at the point upon which it is applied.

In this country true external aneurisms are not common. Your committee are cognizant of but five instances in which the treatment by pressure, as now recommended, has been made use of in the lower extremity. All but one of these have occurred within the past year, and the details of them have been kindly furnished by the operators for this Report.

These cases have been treated by Drs. Buck, Rodgers, and Watson, of New York, Knight, of New Haven, and Mütter, of Philadelphia.

The first case was one of femoral aneurism, in which pressure was fairly tried and did not succeed, and it became necessary at last to resort to the operation by ligature.

The instance which occurred to Dr. Rodgers was that of a negro seaman, aged 47, who, two months before, observed a swelling in the popliteal region, which arose after a fall. The tumour was of the size of a duck's egg, and the symptoms of aneurism were well marked. He entered the New York Hospital, and on the 15th of January, 1847, pressure was made upon the artery near the groin by means of an arterial compressor. This was continued till the 12th of February, but it being found impossible to effect the desired object with it, Dr. Rodgers substituted another, consisting essentially of a metallic plate placed upon the inner side of the thigh over the vessel, having three holes at short intervals through which screws passed, each having at one of their extremities a firm pad, and at the other a projection to which a key was adapted, by means of which pressure could be made upon the femoral artery at the different points. This metallic plate was secured over the artery by means of broad straps attached to a sliding plate of steel, secured in a metallic bar, applied longitudinally to the back of the limb.

Soon after the adjustment of this instrument, it was ascertained that the patient loosened the screw in the absence of his attendant. He was now watched night and day for three days, by the end of which time all pulsation in the tumour was entirely arrested. The swelling gradually subsided to half its original size. The only inconvenience experienced by the patient was a numbness of the limb, upon the first removal of the instrument, but this soon left him, and he was discharged cured on the 13th of April.

The case of Dr. Watson was one of femoral aneurism, treated also at the New York Hospital. The subject of it was an intemperate Irish woman, aged 38. The tumour, which was hard and painful to the touch, had existed for a month, and extended from the upper and inner third of the thigh to within a finger's breadth of the internal condyle of the femur, and at the point of its greatest circumference, reached from the inner border of the rectus muscle to the middle of the outer side of the thigh.

At midday, on the 23d of September, 1847, pressure was made by means of two pads secured to circular straps over the artery, with counter-compresses on the outer side of the thigh, the pressure produced being regulated by a screw which acted directly upon the pads over the vessel. The first compress was fastened over the artery just as it emerges from beneath Poupart's ligament, the second, a short distance below it, and both were so arranged as partially to control the circulation in the tumour, the pressure being regulated by alternately tightening one compress and slackening the other, in order to prevent abrasion of the integument. On the 24th the patient was restless; on the 25th she complained greatly of cramp and pain in the leg, which was much swollen, to relieve which a roller was applied, and the limb elevated on a pillow.

By the 26th all pulsation had left the tumour. The upper compress was now removed—sixty-eight hours having elapsed from its first application, and very slight pressure was kept up by means of the lower one. No return of pulsation followed the removal of the compress, and in an hour afterwards the lower pad was also taken away. The skin beneath the upper compress had become somewhat abraded by the pressure, which required the application of a poultice, and subsequently simple dressings for a few days.

On the 28th no pulsation could be detected in the tumour, or in the femoral artery below the point, upon which the upper pad had rested.

By the 28th of October the tumour had much diminished in size, and become softer.

On the 12th of November, nearly two months subsequent to the commencement of the treatment, she left the hospital well, the tumour still gradually becoming smaller. There was no pulsation to be detected in the anterior or posterior tibial arteries, or at any point below the giving off of the profunda, and the femoral artery itself below that point was felt like a solid cord beneath the integument.

The case furnished by Dr. Mütter was that of a book-keeper, aged 41, whose general health was feeble, and who, six weeks previous to the 24th of September, 1847, had been seized with stiffness in the right ham, which was soon followed by a pulsating tumour of the size of a turkey's egg. After a few days' rest in the horizontal position, his treatment was commenced by applying a roller to the limb in order to prevent swelling, and the application of one of Charrière's compressors, with a small oval pad over the femoral vessel where it passes down to become popliteal, and another similar compressor with a larger pad over the artery at the upper third of the thigh. The limb was then placed upon an inclined plane. After remaining in this position for twelve hours, the lower compressor was tightened until all pulsation ceased in the tumour. The pain produced by this procedure was severe, and could only be borne at first for half an hour. When it became insupportable, the upper compressor was screwed down, and the pressure from the lower one removed. The patient supported the pressure above for two hours without much difficulty; it then became annoying, and in order to relieve the suffering, the lower compressor was again tightened.

By thus alternating the points to which it was applied, the necessary amount of pressure was kept up without excoriation, or any other injurious consequence resulting, and from the peculiar construction of the instruments, and the previous application of the roller, the swelling of the limb was trifling. During the treatment, the diet of the patient was restricted, and digitalis was administered to him.

By the twelfth day the tumour was reduced to about half its original size; had become solid, and was free from pulsation. Notwithstanding these circumstances, Dr. Mütter did not consider it safe to allow his patient to move about, or even to relax the treatment, but continued to pursue the same course, with slight modifications, for six weeks longer. At the expiration of this period, the tumour had nearly disappeared, the collateral circulation was fully established, and the disease radically cured.

Dr. Knight's case, which is peculiarly interesting, from the novel manner in which the pressure was made, and quickly effected a cure, was that of a mulatto man, aged 48, in whom a popliteal aneurism had existed for several months. The aneurismal tumour, which was well marked, filled up the whole popliteal space. The leg was very painful and œdematous. After the œdema was removed by rest, and other appropriate treatment, pressure on the artery, by means of the hoop tourniquet, the calliper shaped instrument, the common tourniquet, guarding the limb against pressure of the strap by encasing it with thick sole-leather, and by a variety of other mechanical contrivances, was fairly tried. By whatever instrument, however, the pressure was made, and however carefully it was guarded, whether continued on one point only, or shifted from one part of the artery to another, the pain became in a short time so severe that it could not be endured. The pain complained of was not in the part pressed upon by the instruments, but was felt equally in the thigh and below the knee, and occurred whether the limb was left uncovered or was enveloped in a roller. It usually began in twenty-five or thirty minutes after the pressure was made, and became intolerable in fifteen or twenty minutes longer, and could be continued in no instance beyond one hour. These efforts were persisted in for eight or ten days, and as nothing had been gained at the end of that time, were abandoned. Before resorting, says Dr. Knight, to the ligature of the artery, I concluded, with the concurrence of his physician, Dr. Tyler, to try manual pressure upon the vessel. "To accomplish this, a sufficient number of assistants were procured from the members of the medical class, who cheerfully offered their services. They were divided into relays, two keeping up the pressure for five or six hours, relieving each other every hour or half hour, and these succeeded by two others. Sufficient pressure to arrest the pulsation in the tumour was found to be most easily made with the thumb or fingers, without a compress, upon the artery as it passes over the os pubis, and the direction given to the assistants was to keep up this amount of pressure as nearly continuously as possible." This treatment was commenced at 3 o'clock, P. M. No pain of consequence was produced by it for five or six hours, and then it was not severe, and was quieted by the eighth of a grain of morphia once or twice repeated. About eight hours after the pressure was applied, the temperature of the limb was diminished, and it appeared shrunken in size. Upon removing the pressure from the artery at 11 o'clock of the following day—twenty hours from the commencement of the treatment, the tumour was found to have diminished very little, if at all, and pulsated as strongly as before; but the tibial arteries could not be felt. The treatment was continued. Upon examining the parts the next morning, forty hours after the treatment was begun, the tumour was found to be nearly one-third less in size, firm and unyielding on pressure, and entirely without pulsation. All treatment was then discontinued. The femoral artery pulsated with its usual strength in the groin, and distinctly as far as its passage through the tendon of the adductor muscles. Between this point and the tumour it could not be felt. Several of the anastomosing arteries, especially one upon the inside of the limb, could be distinctly traced passing over the knee, pulsating strongly, and enlarged in size. From that time to the present—a period of more than four months—no change has taken place in the limb, except that the tumour has gradually diminished, so as now to be scarcely discoverable, and that the leg, which was at first cold and weak, has nearly regained its natural temperature and strength.

Within the past year another instance has been reported, in which a ligature was placed upon the common iliac artery for aneurism, by Dr. Lyon, of England. The bold and successful precedent for this, it will be remembered, was first done by Dr. Mott, of New York, in 1827. In 1812 the common iliac was tied, in a case of gun-shot wound, by Dr. Gibson The patient lived thirteen days after the operation. Since this period, the case just alluded to makes the eighteenth in which the operation has been performed, of which eight have proved successful, and ten have died. The aim of this Report being to show results, rather than to give the details of operative procedures, particularly when not done among us, a bare mention of this fact will suffice. In connection with it, it may be interesting to notice the final issue of the instance in which this artery was last tied in this country. The operation was done by Dr. Peace, at the Pennsylvania Hospital, and the details of it published in the *American Journal of Medical Science*, for 1843. The subsequent history of it now given, has been furnished to the committee by the operator. The artery was tied August 29th, 1842, and the patient was discharged cured on the 8th of October. Five months after it was done, the tumour, which had been very large, was found to be hard, was greatly reduced in size, and continued free from pulsation. He returned to a laborious occupation, and in November, 1843—fifteen months after the operation—his attention was directed to a re-appearance of the tumour. He now presented himself to his surgeon, who found it soft, fluctuating, and of the volume of a small orange, with the integuments covering it discoloured. He was re-admitted into the hospital, and in a few days ulceration took place, and he died after repeated hemorrhages. After his admission, ligature of the aorta was suggested, with a view of prolonging life, but it was found that pressure on this great trunk at its lower part did not arrest the flow of blood, and was, of course, abandoned. The pelvis, which was bequeathed to Dr. Peace, is now in his possession, and shows that the ligature had been placed upon the iliac vessel just above its bifurcation—a point at which it was perfectly sound—and that the hemorrhage was due to the return of blood into the tumour by the collateral vessels. These vessels being given off by the aorta, above the point at which it is prominent upon the vertebræ, and where it had been compressed in the examinations which were made.

That a ligature may be placed upon the aorta, there are recorded observations to attest: that it will ever be followed by any lasting benefit, there is every reason to doubt. Cooper's patient having died in forty; James' in three, and Murray's in twenty-three hours after it was done. As adding, however, to the list of cases, which show that the collateral vessels are fully able to carry on vigorously the circulation after its complete obliteration, a case which has been detailed by Dr. West, in the No. of the *Transactions of the Philadelphia College of Physicians* for February of the present year, is worthy of notice. The subject of it, who was aged 32, and died suddenly from the rupture of an internal aneurism, was remarkably muscular and athletic, with the superior half of his body more developed than the lower. The interesting feature of the case, for our purposes, was, that in tracing the aorta beyond the origins of the great vessels, its cavity was found to be *entirely obliterated* immediately beyond the ductus arteriosus. At the point of obliteration, it presented a well defined and regular contraction, which looked as if it had been produced by a ligature thrown around the artery. Beyond this, the vessel resumed very nearly its natural dimensions, and so continued throughout its course. It gave origin, in its whole length, to the usual branches; the upper pair of intercostals coming off immediately below the stricture. The internal mammary arteries, which pursued their course along the thoracic parietes in a very tortuous manner, were fully as large as the internal iliacs, and so were the epigastrics; these vessels constituting the main channels for keeping up the connection of the circulation above and below the aortic stricture.

From answers that have been received to the circular letter of the committee, some facts have been communicated for the Association, which they will now endeavour to give, as far as possible, in the language of their correspondents. Dr. March, of Albany, describes what he believes to be an easy and prompt mode of effecting the radical cure of hydrocele. His operation consists in using a pretty large sized round trocar, by which the puncture is made, and through the canula of which the water is permitted to flow out. The canula is to be kept still in the sac, and through it a camel's hair pencil, dipped into a solution of the iodide of potassium is to be passed, and the whole inner surface of the tunic painted over, which may require the pencil to be armed with the solution three or four times. He adds, that he has operated in this way in a number of instances with complete and most satisfactory success.

Dr. Horner, of Philadelphia, in his communication to the committee remarks, that he has found the treatment of hydrocele rendered much more certain by the introduction of a few threads passed through the tunica vaginalis from the bottom to the top, even where the process by injection is the main feature of the case. His experience has shown him that no injection is to be wholly relied on, unless in connection with subsequent treatment. The effect of any irritating injection into the tunica vaginalis, he observes, is not only to produce a secretion of lymph, but also of serum, and if the latter accumulates, it will of course keep separated the opposed sides of the tunica vaginalis, hence a common cause, perhaps the most frequent, of failure in all injections. This obstacle, he adds, may be very easily overcome with four or five threads of silk, which will carry off the serum as fast as it is secreted, and thus allow the two surfaces of plastic membrane to touch and coalesce.

Dr. Brainard, of Chicago, has forwarded for the consideration of surgeons, some practical remarks in reference to the treatment of extensive suppuration by astringent and stimulant injections into the suppurating cavity. In these cases, free openings, and washing the surface within with a solution of ℥ij of alum, and ℥i of sulphate of copper to the pint of water, will check its progress, diminish the quantity, and improve the quality of the pus. He is inclined also to think that the practice prevents purulent absorption, and by exciting inflammation, limits its spread among the tissues. The same application he has found useful on the surface of large stumps where the pus is abundant and offensive.

Dr. Brainard also advises us that he has lately used iodine injections in serous effusions. "Having noticed," he says, "the rapidity with which patients often sunk after puncture of ascites, spina bifida, &c., I formed the opinion that such treatment was wrong, and determined to try injections. Having occasion soon after to treat a case of spina bifida, in the Chicago Hospital, in a child about 13 years old, in whom the tumour was situated at the top of the sacrum, being nine inches in circumference, and about three inches in height, with thin walls, I determined to inject into the sac a solution of iodine, with a view of exciting inflammation and procuring absorption. This was done on the 2d December, 1847, in the following manner. A small puncture was made with a lancet half an inch from the base of the tumour, and a trocar of the size of a knitting needle carried obliquely into the sac. Through the canula of this, a solution of gr. i. of hydriodate of potash, with gr. ss. of iodine, in a fluid-drachm of water, was thrown into the sac, and the instrument withdrawn without allowing the serum to escape. A sharp pain followed, which soon subsided, and compresses were applied to prevent the escape of fluid. After the operation the tumour became red, tense, and tender; but these symptoms soon gave place to a remarkable flabbiness and contraction. By the 27th of December it had diminished to about half its former size. At this date a second injection was used of half the strength of the first, which produced but little heat or pain, and the compression was continued. On the 15th January, 1848, so much of the fluid was absorbed as to render it easy to press it down almost to a level with the surrounding parts, the skin was lying in wrinkles over it, and the bony opening could be distinctly felt. Since the last date a third injection has been made without any unpleasant result. Dr. Brainard thinks that the injection of a solution of iodine, (so far as a single case can be taken as a guide,) is attended with but little danger, and may be capable of curing hydrorachitis.

Dr. Jno. C. Warren writes, that in the treatment of fractures of the condyles of the os humeri, a course is usually recommended which he believes to be hurtful, inasmuch as it favours the worst consequence of the injury, namely, loss of motion in the joint. The common practice, he observes, in the treatment of these cases, is to apply angular splints destined to prevent motion, and in about two weeks, to make passive movements for the purpose of preventing the adhesion of the fractured portions in such a manner as to impede the free action of the joint. By this mode of treatment, the fractured piece becomes sufficiently fixed to create partial anchylosis; and there is so much pain afterwards in the proposed passive movements, as to cause the omission of these measures, until permanent stiffness takes place. The proper course in the management of these accidents, he conceives, to be—1st. To apply no splints, but in the earlier days to make use of the proper means to prevent inflammation. 2d. To accustom the patient to early and daily movements of flexion and extension. 3d. When the action of the joint becomes limited, to overcome the resistance by force, and repeat it daily until the tendency of the joint to stiffen ceases.

The accomplishment of this process, he adds, is so very painful, that few patients have courage to submit to it, and few surgeons firmness to prosecute it. The consequence has been, that in a great number of cases the use of the articulation to a greater or less extent has been lost. The introduction of etherization by preventing the pain, gives us, in the opinion of Dr. Warren, the means of overcoming the resistance. By its aid, he has restored the motion of a considerable number of anchylosed elbows, and has successfully applied the same measures to other joints, particularly to the shoulder and knee. This has now become his settled practice, with the results of which he is entirely satisfied. The inflammation consequent upon the forced movements of an anchylosed joint, is not to be lost sight of. By a reasonable abstraction of blood and other anti-inflammatory treatment, he has never found it alarming.

Within a year or two past, attention has been, in a particular manner, directed to derangement of the cerebral functions following ligature of the common carotid artery. These cerebral symptoms are attributable either to cutting off the direct supply of blood to the brain, or to disease consequent upon the altered condition of the circulation in that organ. Nearly one-fifth of the recorded cases of the operation in question, are found to have exhibited it in a greater or less degree; and the frequency of its occurrence has been singularly overlooked by practical surgeons. Two cases have been forwarded to the committee by Dr. Mettauer, of Virginia, in which it was observed: in these the vessels were taken up, in one instance, for an anastomosing aneurism of the antrum and nasal cavities, and in the other for the cure of a false aneurism. Both patients had lost large quantities of blood previous to the operations. In each case partial hemiplegia of the opposite side to the artery which was ligatured, was noticed in a few hours, and was followed by delirium and convulsions. In one of the instances, death occurred on the 8th, and in the other on the 10th day.

Autopsic examinations showed softening of the medullary substance on the side opposite to that on which the vessel was tied, while the hemisphere corresponding to it was healthy, though pale and bloodless.

